# New Insights into the Occurrence and Toxin Profile of Ciguatoxins in Selvagens Islands (Madeira, Portugal)

**DOI:** 10.3390/toxins10120524

**Published:** 2018-12-07

**Authors:** Pedro Reis Costa, Pablo Estevez, David Castro, Lucía Soliño, Neide Gouveia, Carolina Santos, Susana Margarida Rodrigues, José Manuel Leao, Ana Gago-Martínez

**Affiliations:** 1 IPMA—Portuguese Institute of the Sea and Atmosphere, Av. Brasília, 1449-006 Lisbon, Portugal; prcosta@ipma.pt (P.R.C.); lucia.solino@ipma.pt (L.S.); srodrigues@ipma.pt (S.M.R.); 2 Faculty of Chemistry, Department of Analytical and Food Chemistry, University of Vigo, Campus Universitario de Vigo, 36310 Vigo, Spain; paestevez@uvigo.es (P.E.); dcastro@uvigo.es (D.C.); leao@uvigo.es (J.M.L.); 3 Regional Fisheries Management—Madeira Government, DSI-DRP, Estrada da Pontinha 9004-562 Funchal, Madeira, Portugal; neide.gouveia@madeira.gov.pt; 4 Instituto das Florestas e Conservação da Natureza, IP-RAM, Secretaria Regional do Ambiente e Recursos Naturais, Regional Government of Madeira, IFCN IP-RAM, Quinta Vila Passos—Rua Alferes Veiga Pestana 15, 9054-505 Funchal, Madeira, Portugal; carolinasantos@gov-madeira.pt

**Keywords:** ciguatera fish poisoning, gambierdiscus, seafood safety, C-CTX-1

## Abstract

Ciguatoxins (CTXs), endemic from tropical and subtropical regions of the Pacific and Indian Ocean and the Caribbean Sea, have caused several human poisonings during the last decade in Europe. Ciguatera fish poisonings (CFP) in Madeira and Canary Islands appear to be particularly related with consumption of fish caught close to Selvagens Islands, a Portuguese natural reserve composed of three small islands that harbor high fish biomass. In this study, fish specimens considered as potential vectors of CTXs were caught in Madeira and Selvagens archipelagos for toxins determination via sensitive liquid chromatography with tandem mass spectrometry detection (LC–MS/MS). CTXs were found in most of the fish samples from Selvagens and none from Madeira. Caribbean ciguatoxin-1 (C-CTX1) was the only toxin congener determined, reaching the highest value of 0.25 µg C-CTX1 kg^−1^ in a 4.6 kg island grouper (*Mycteroperca fusca*). This study indicates that a diversity of fish from different trophic levels contains CTXs, Selvagens appear to be one of the most favorable locations for CTXs food web transfer and finally, this study highlights the need of further research based on intensive environmental and biological sampling on these remote islands.

## 1. Introduction

Ciguatera fish poisoning (CFP) is a human food-borne illness caused by consumption of tropical and subtropical fishes harboring ciguatoxins (CTXs). Coral reef fish are frequently reported to accumulate CTXs, which are products of fish metabolism resulting from biotransformation of precursor compounds produced by the benthic dinoflagellates, *Gambierdiscus* and *Fukuyoa*, and transferred through the marine food web [1,2,3]. 

CTXs have been classified according to their geographic distribution through tropical regions, as Pacific (P), Caribbean (C) and Indian Ocean (I) CTXs-group toxins. CTXs are lipophilic polyether compounds consisting of 13 or 14 ether rings forming a ladder-like structure, being known up to 40 CTXs congeners [4]. CTXs are highly potent neurotoxins that act at voltage gated sodium channels (VGSC) increasing sodium ion permeability and cell disruption [5,6]. Humans affected by CTXs revealed a wide range of gastrointestinal (abdominal pain, nausea, vomiting, diarrhea), neurological (allodynia—burning pain caused by cold stimulus—, parestesia, dysesthesia) and cardiovascular symptoms (bradycardia, hypotension) [7,8]. The neurological symptoms may persist from weeks to months, or in cases without the appropriate treatment may become chronic [9]. In severe cases, CTXs can be fatal [10]. 

Possibly related to structural differences of CTXs congeners among regions, differences in human symptoms have been observed in outbreaks occurring in the Caribbean, Indian and Pacific Ocean. While neurological symptoms are highly dominant in the Pacific, gastrointestinal disorders are frequently reported in the initial phases of intoxications in the Caribbean, which may then be followed by neurological symptoms. In the Indian Ocean, neurological symptoms have also included hallucinations [11,12].

In Europe, CFP has been mainly seen as an issue of travellers to endemic regions, or resulting from consumption of toxic fish imported from tropical areas [13,14]. Therefore, legal framework in the EU is scarce, without regulatory limits imposed for CTXs-group toxins. In order to prevent the imports of toxic fish from endemic regions the EU legislation states that fishery products containing biotoxins, such as ciguatoxin or muscle-paralysing toxins must not be placed on the market [15,16]. Relatively recently, a paradigm shift had occurred after several human CFP outbreaks in the Spanish and Portuguese Atlantic islands of Canary, Madeira and Selvagens archipelagos. In 2004, five individuals suffered from gastrointestinal and neurological disorders, consistent with CFP, after consumption of amberjack *Seriola rivoliana*, weighing 26 kg, captured by scuba divers along the coast of the Canary Islands [17]. The first outbreak in Portugal occurred in 2008, when 11 crew members of a fishing boat reported CFP symptoms after consumption of amberjack (30 kg) caught around Selvagens Islands [18,19]. In the same year, 20–30 people reported CFP symptoms after consuming amberjack purchased in the markets of the Canary Islands but caught close to Selvagens Islands [20]. 

Subsequent episodes led local and European authorities to take into consideration a new emerging risk of food poisoning. A monitoring program for detection of CTXs by cell based assays was implemented in the Canary Islands and restrictions to fisheries have been implemented in Madeira and Selvagens archipelagos. A panel of experts of the European Food Safety Authority (EFSA) stated a scientific opinion on the risk to human health related to the consumption of ciguatoxins in fish and emphasized the need for more data including data on the occurrence of CTXs in fish from European waters [21]. In addition, more recently a pan-European project was co-funded by EFSA to improve the risk characterization of CFP in Europe [22].

This study aims to (1) investigate the presence of CTXs in Portuguese waters by comparing CTXs occurrence in fish caught in Madeira and Selvagens Islands, and (2) to determine the CTXs profile in contaminated fish by sensitive liquid chromatography with tandem mass spectrometry detection (LC–MS/MS).

## 2. Results

**Figure 1:**
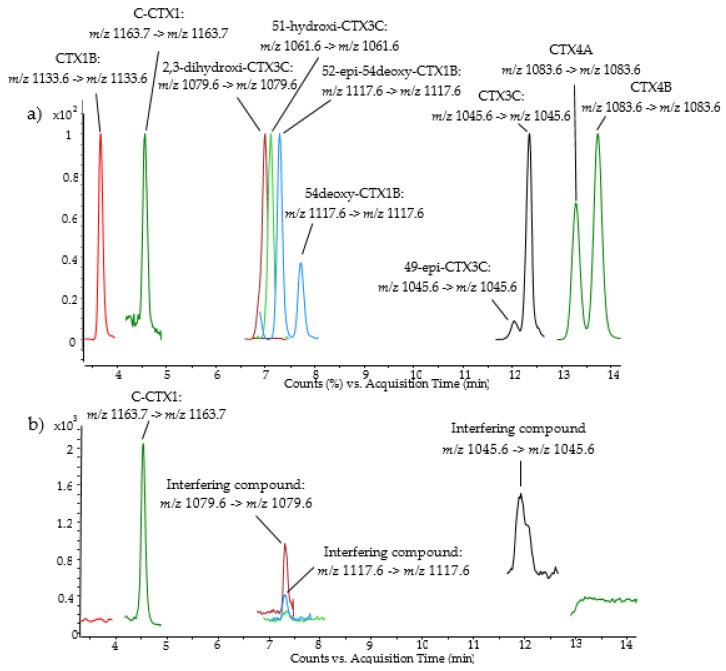
Sensitive liquid chromatography with tandem mass spectrometry detection (LC–MS/MS) chromatogram in Multiple Reaction Monitoring (MRM) mode of (**a**) a mixture of P-CTXs and C-CTX1 standard solution, and (**b**) CTXs profile in a sample of fish tissue (Island Grouper) from Selvagens Islands.

Caribbean ciguatoxin-1 (C-CTX1) was detected by LC–MS/MS in eight out of 11 samples from Selvagens Islands while no CTXs were detected in any of the samples analyzed from Madeira Island. An example of the results obtained is shown in Figure 1. The retention time and transitions *m*/*z* 1163.7 -> *m*/*z* 1163.7 of C-CTX1 precursor -> product ion in the evaluated samples were consistent with the ones found for the pure standard of C-CTX1. Some interfering peaks were also detected, which could be associated to Pacific ciguatoxins (P-CTXs) based on the similar *m*/*z* and precursor/product ion transition, nevertheless, the retention times did not match with the ones found for the standard solutions of these P-CTXs analogues. 

C-CTX1 was detected in fish of different size ranges and different trophic levels (Table 1). Due to the limited amount of C-CTX1 pure standard, C-CTX1 content was expressed in CTX1B equivalents and samples where C-CTX1 was detected were quantified as CTX1B and converted to C-CTX1 equivalents taking into account the conversion factor previously obtained for the pure standard of C-CTX1. Calibration was therefore carried out by using the pure standard of CTX1B in the concentration range of 0.28–27.88 ng mL^−1^ (*n* = 5, *R*^2^ = 0.999). Limits of detection (S/N >3) and quantitation (S/N >10) were of 0.0045 and 0.0150 ng g^−1^ in matrix matched samples spiked with CTX1B standard solution. The highest concentration level, reaching 0.25 µg C-CTX1 kg^−1^, was determined in a 4.5 kg Island Grouper (sample 4), followed by a 1.6 kg Barred Hogfish (sample 6), which contained 0.11 µg C-CTX1 kg^−1^. The toxin concentration determined in the >19 kg top predator and known vector of CTX, the Dusky Grouper (sample 1), only accounted for a toxin concentration of 0.05 µg C-CTX1 kg^−1^.

## 3. Discussion

Several outbreaks reported in the Canary Islands since 2004 derived from consumption of fish caught close to the Selvagens Islands [20]. The cause of CFP in Portuguese waters has also been related to contaminated fish from Selvagens [18,19]. In the present study, CTXs was not detected in samples caught along the Madeira Island coast but were detected in most fish samples obtained from the further south Selvagens Islands. With the data available at the present day, reinforced with results of this study, one may argue that Selvagens islands are a hotspot of CTXs in Europe. 

In this study, Caribbean CTXs was detected not only in top predators, as is the big size >19 kg dusky grouper, *Epinephelus marginatus*, but also in smaller sized fish from intermediate levels of the trophic chain. The highest concentration was registered in a 4.5 kg Island grouper (*Mycteroperca fusca*). This species is a near rocky or sandy-rocky sea-beds inhabitant that preys on fish, crustaceans, and cephalopods. Its abundance has been positively correlated with upright seaweed cover, and individuals may reach a longevity of 30 to 40 years and up to 7.8 kg [23]. It is common to associate higher CFP risk with larger fish and indeed, fishing amberjacks in Madeira is restricted to specimens not exceeding 10 kg. However, in addition to the fish size, longevity should also be considered. While island grouper may not reach a similar high size/weight as amberjacks, this species may live longer to accumulate high CTXs levels. In fact, after the first report of CFP in Portugal, due to consumption of 30 kg amberjack by crew members of a fishing boat, CFP was retrospectively identified in nature wardens of Selvagens Islands Nature Reserve that consumed parrotfish (*Sparissoma cretense*), blacktail comber (*Serranus atricauda*), barred hogfish (*Bodianus scrofa*), grey triggerfish (*Balistes capriscus*) and red porgy (*Pagrus pagrus*) and suffered from neurological disorders between 2 and 6 weeks [18]. However, LC–MS/MS analyses for the detection of CTXs had never been carried out in these fish species from Selvagens until the present study.

While several CTXs congeners have been indicated by [19] in fish caught from Selvagens after the 2008 incident, in the present study only C-CTX1 was detected, which is in agreement with the toxin profile described by [20] during outbreaks in Canary islands and it is typically observed in the Caribbean Sea [24]. Further studies need to be carried out to confirm the presence of other CTXs congeners, having in mind that LC–MS/MS analysis of CTXs is complex and interfering compounds leading to misidentification may occur. This fact was observed in some samples of pink dentex from Madeira (samples M3 to M5), which contained an interfering compound that was initially misidentified as a potential CTXs analogue.

According to EU directives, there are no limits for CTXs in fish, but the presence of CTXs as determined by any detection method is enough to ban the fishery products from the markets [15,16]. However, the toxic potential of C-CTX1 is assumed to be lower than most of other CTXs congeners, in particular to those from Pacific Ocean. EFSA stated that until better data is available, the following toxicity equivalency factors (TEFs) should be used to express fish toxicity as CTX1B equivalents: CTX1B = 1 and C-CTX1 = 0.1 [21]. EFSA estimates that fish containing 0.1–5 µg CTX1B kg^−1^ has been related with cases of CFP and that a concentration of 0.01 µg CTX1B kg^−1^ of fish is a level expected to not exert effects in sensitive consumers. In the present study, the highest concentrations reached in sample S4 and S6 are, according to EFSA, as well as according to the guidance levels established by the Food and Drug Administration (FDA) of the USA, low and around the safety levels respectively.

## 4. Conclusions

The evaluation of the risk of CFP in Europe is a very challenging task for scientists, seafood safety authorities and environmental managements. There are still unresolved issues related to the toxicology of the different CTXs analogues and metabolites, as well as with analytical methods, in particular due to the lack of reference materials, seasonal and spatial variability of toxin vectors and of the toxin precursor producers.

This study highlights Selvagens Islands as a key location to carry out studies on CFP incidence as an example of the emergence of this toxin in the EU coasts. Indeed, Selvagens may be used as a sentinel site for comparative CTXs occurrence in the Canary Islands and even Madeira. Selvagens Islands are a healthy ecosystem where fishing pressure is minimal, thereby fish may grow and reach high longevity and consequently harbor greater levels of CTXs. However, further studies involving intensive sampling in these remote Islands should be carried out to understand the *Gambierdiscus* dynamics, the toxin transfer in the food web and fish toxin metabolism.

## 5. Materials and Methods 

### 5.1. Madeira and Selvagens Islands 

**Figure 2:**
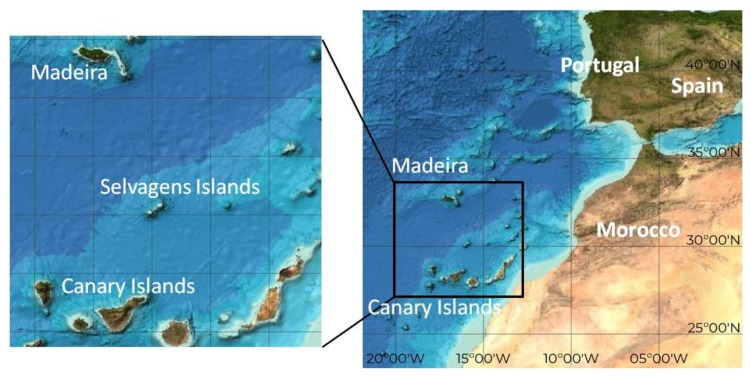
Location of Selvagens Islands (Madeira, Portugal).

Selvagens Islands are a very remote and isolated group of Portuguese oceanic islands, located in the Northeastern Atlantic, 293 km southeast from Madeira Island, Portugal, 180 km north from Tenerife Island, Spain and 600 km west from the African coast (Figure 2). They are comprised of three islands of volcanic origin: Selvagem Grande, Selvagem Pequena and Ilhéu de Fora, and several islets, shaped specially by marine abrasion. Classified as a nature reserve in 1971, with a total marine area of 92 km^2^ and included within the Natura 2000 Network, with a total marine area of 1242 km^2^, the biodiversity of the Selvagens Islands has greatly benefitted from this protection and the successive conservation action, being an example of a high coastal species diversity occurring even in very small areas of the northeastern Atlantic Ocean.

Selvagens have been designated by National Geographic Society as one of the pristine sites in the oceans today [25]. Human pressure is very low in Selvagens, where limited number of visitors is allowed each year. In contrast, Madeira Island is heavily populated and a high popular tourism destination. As a result of the remote location of Selvagens Islands and the high anthropogenic pressures at Madeira, total fish biomass was estimated to be 3.2 times higher at Selvagens than at Madeira, and when considering only the fish top predators biomass it reach values 10 times higher at Selvagens [26]. Previous studies describing the marine fish diversity found that 34.1% of the ichthyofauna observed in Selvagens also occurs in the Canary Islands and 47.3% in Madeira Island [27].

### 5.2. Fish Samples

Specimens of fish species previously identified as vectors or potentially vectors of CTXs and representative of different trophic levels from both Madeira and Selvagens Islands were opportunistically obtained for this study (Table 2). A portion of fish flesh (>100 g) were dissected from muscle close to fish head and stored at −20 °C until analysis.

### 5.3. Reagents

Acetone, diethyl ether, methanol, water, hexane and ethyl acetate used for ciguatoxins extraction and purification were of HPLC grade (Merck KGaA, Darmstadt, Germany). Methanol, formic acid, ammonium formiate (Merck KGaA, Darmstadt, Germany) and water (J. T. Baker, Center Valley, PA, USA) were of LC–MS grade.

Pure standard solution of CTX1B, a mixture of Pacific Ciguatoxins standard solution containing CTX1B, 2,3-dihydroxi-CTX3C, 51-hydroxi-CTX3C, 52-epi-54-deoxy-CTX1B/54-deoxy-CTX1B, 49-epi-CTX3C/CTX3C, CTX4A/CTX4B were kindly supplied by Prof. Takeshi Yasumoto (Japan Food Research Laboratories). C-CTX1 pure standard solution was kindly provided by Dr. Robert Dickey (previously, U.S. Food and Drug Administration) via Dr. Ronald Manger (Fred Hutchinson Cancer Research Center, Seattle, USA).

### 5.4. Ciguatoxins Extraction

CTXs sample pretreatment was carried out following the conditions proposed by [29] with modifications [30,31], briefly: Fish flesh samples (15 g) were extracted twice by homogenizing 2 min at 9000 rpm in 45 mL of acetone (Ultra Turrax^®^ T25 basic IKA^®^ WERKE, Staufen, Germany). The combined extracts were concentrated to an aqueous residue and extracted twice with 15 mL of diethyl ether and further evaporated to dryness. The organic residue was dissolved in 4.5 mL 90% MeOH and defatted with 9 mL of hexane evaporating the aqueous layer using a multievaporator under reduced pressure (Syncore^®^ Polyvap, Barcelona, Spain). The remaining solid residue was dissolved in 2 mL of ethyl acetate and further purified through a solid phase extraction (SPE) cleanup including two different SPE mechanisms. The first normal phase Florisil SPE was used to remove polar interferences whereas non-polar and semipolar interferences were removed by using C18 SPE reverse phase. Cleanup conditions are described as follows: The 2 mL of ethyl acetate extract from extraction were passed through a Florisil cartridge (J. T. Baker, 500 mg, Center Valley, PA, USA) conditioned with 3 mL of ethyl acetate, and washed consecutively, with 3 mL of ethyl acetate and 5 mL of ethyl acetate-methanol (9:1) and ethyl acetate-methanol (3:1) [32]. Previous work carried out by this group on the optimization on the sample pretreatment for the LC–MS/MS analysis of CTXs allowed to conclude that the toxin mainly elutes in the second fraction with a recovery around 80% [30]. The residue containing the toxin was further dried under nitrogen at 50 °C and then dissolved in 2 mL of MeOH 60% and applied to a C18 cartridge (SUPELCLEAN, Supelco, 500 mg, Bellefonte, PA, USA) conditioned with 3 mL of MeOH 60%. The cartridge was washed with 3 mL of MeOH 60% and CTXs were eluted with 5 mL of MeOH 90%. The final eluate was dried and dissolved in 0.5 mL of MeOH LC–MS grade filtering (Syringe Driver filter Unit, Millex^®^-CV 0.22 um, 13 mm, Millipore, Billerica, MA, USA) prior to LC–MS analysis.

### 5.5. LC–MS/MS Analysis

LC–MS/MS analysis was carried out following [29] conditions with modifications introduced in the LC–MS instrument in order to improve sensitivity [30,31]. LC–MS/MS analyses were performed by using an Agilent 1290 Infinity LC system coupled to an Agilent 6495 Triple Quadrupole LC–MS (Agilent Technologies, Waldbronn, Germany) equipped with an Agilent Jet Stream electrospray ionization source (iFunnel).

Analytes were separated in a Poroshell 120 EC-C18 (3.0 × 50 mm, 2.7 µm, Agilent Technologies, Waldbronn, Germany) with column temperature set at 40 °C. LC mobile phase was: 5 mM ammonium formate and 0.1% formic acid in water (A) and MeOH (B). Gradient used was 78% B to 88% B in 10 min and held for 5 min, increased to 100% B at 15.01 min and held 3 min returning to 78% B at 18 min, and 4 min of equilibration before the next injection. The injection volume was 1 µL and the flow rate 0.4 mL/min. The mass spectrometer was operated in positive mode monitoring [M+Na]^+^ as precursor and product ions with collision energy of 40 eV. This approach allows a sensitive detection of the CTXs compared to other strategies that monitor CTXs water losses [33,34]. The selection of a single stable [M+Na]^+^ as precursor ion, with high collision energy and methanol as mobile phase, allows the removal of the background noise monitoring the same [M+Na]^+^ as product ion without any additional fragmentation due to the high stability of this adduct under the above mentioned conditions.

LC–MS/MS system settings were: Drying gas, 15 L min^−1^ of N_2_ at 290 °C; sheath gas flow, 12 L min^−1^ of N_2_ at 400 °C; nebulizer gas, N_2_ at 50 psi; capillary voltage, 5000 V; nozzle voltage: 300 V; fragmentor potential 380 V. CTXs were monitored by MRM as follows: CTX1B (*m*/*z* 1133.6 -> *m*/*z* 1133.6), C-CTX1 (*m*/*z* 1163.7 -> *m*/*z* 1163.7), 2,3-dihydroxi-CTX3C (*m*/*z* 1079.6 -> *m*/*z* 1079.6), 51-Hydroxi-CTX3C (*m*/*z* 1061.6 -> *m*/*z* 1061.6), 52epi-54deoxy-CTX1B/54deoxy-CTX1B (*m*/*z* 1117.6 -> *m*/*z* 1117.6), 49-epi-CTX3C/CTX3C (*m*/*z* 1045.6 -> *m*/*z* 1045.6), CTX4A/CTX4B (*m*/*z* 1083.6 -> *m*/*z* 1083.6).

## Figures and Tables

**Table 1:** Concentration of ciguatoxins (C-CTX1) determined in fish flesh from Selvagens Islands.

Sample ID	Species	Common Name	C-CTX1 (µg kg^−1^)
S1	*Epinephelus marginatus*	Dusky grouper	0.05
S2	*Bodianus scrofa*	Barred hogfish	<LOQ
S3	*Balistes capriscus*	Grey triggerfish	<LOQ
S4	*Mycteroperca fusca*	Island grouper	0.25
S5	*Serranus atricauda*	Blacktail comber	<LOQ
S6	*B. scrofa*	Barred hogfish	0.11
S7	*B. scrofa*	Barred hogfish	0.06
S8	*B. capriscus*	Grey triggerfish	0.03
S9	*Kyphosus sectatrix*	Bermuda sea chub	<LOD
S10	*K. sectatrix*	Bermuda sea chub	<LOD
S11	*Sphyraena viridensis*	Yellowmouth barracuda	<LOD

LOQ Limit of quantification (S/N >10): 0.0150 ng·g^−1^; LOD Limit of detection (S/N >3): 0.0045 ng·g^−1^.

**Table 2:** Biological and biometric parameters of the fish sample obtained from Selvagens and Madeira Islands for CTXs analysis.

Sample ID	Species	Common Name	Feeding Habits (H/C) ^‡^	Weight (g)	Total Length (mm)	Fork Length (mm)	Gender (Maturity Stage ^§^)	Capture Date (mm/dd/yy)
	**Selvagens Islands**							
S1 *	*Epinephelus marginatus*	Dusky grouper	C	19,500	970	-	-	12/13/16
S2 *	*Bodianus scrofa*	Barred hogfish	C	2362	510	-	F (-)	12/13/16
S3 *	*Balistes capriscus*	Grey triggerfish	C	2208	500	462	M (-)	12/13/16
S4 *	*Mycteroperca fusca*	Island grouper	C	4533	690	657	M (-)	12/13/16
S5*	*Serranus atricauda*	Blacktail comber	C	810	380	-	F (-)	12/13/16
S6	*B. scrofa*	Barred hogfish	C	1652	440	-	-	11/28/17
S7	*B. scrofa*	Barred hogfish	C	770	350	-	F (2)	11/28/17
S8	*B. capriscus*	Grey triggerfish	C	1966	444	425	F (5)	11/28/17
S9	*Kyphosus sectatrix*	Bermuda sea chub	H	2286	531	477	F (5)	11/28/17
S10	*K. sectatrix*	Bermuda sea chub	H	492	315	277	F (2)	11/28/17
S11	*Sphyraena viridensis*	Barracuda	C	1564	-	-	M (5)	11/28/17
	**Madeira Island**							
M1	*Seriola rivoliana*	Longfin yellowtail	C	22,644	1234	1080	M (5)	01/19/17
M2 *	*Makira nigricans*	Blue marlin	C	298,000	-	3150	-	01/06/17
M3	*Dentex gibbosus*	Pink dentex	C	7539	850	751	M (5)	10/12/17
M4	*D. gibbosus*	Pink dentex	C	7841	859	768	M (5)	10/12/17
M5	*D. gibbosus*	Pink dentex	C	8097	846	745	F (5)	10/12/17
M6	*Seriola dumerili*	Greater amberjack	C	20,117	1232	1090	M (5)	10/12/17
M7	*S. dumerili*	Greater amberjack	C	31,480	1390	1221	M (5)	10/12/17
M8	*Sphyrna zygaena*	Smooth hammerhead	C	10,297	1360	-	M (-)	10/12/16
M9*	*Isurus oxyrinchus*	Shortfin mako	C	67,000	-	-	-	06/10/16

* provided by the maritime authority, confiscated due to illegal fishing. ^‡^ H—herbivorous; C—carnivorous. ^§^ Maturity scale adapted from [28].
